# Inhibitory Effect of Triterpenoids from *Panax ginseng* on Coagulation Factor X

**DOI:** 10.3390/molecules22040649

**Published:** 2017-04-24

**Authors:** Lingxin Xiong, Zeng Qi, Bingzhen Zheng, Zhuo Li, Fang Wang, Jinping Liu, Pingya Li

**Affiliations:** 1 School of Pharmaceutical Sciences, Jilin University, Fujin Road 1266, Changchun 130021, China; xionglx14@mails.jlu.edu.cn (L.X.); qizeng95@163.com (Z.Q.); zhengbz15@mails.jlu.edu.cn (B.Z.); rockefeller1988@sina.com (Z.L.); 2 National and Local Joint Engineering Research Center for Ginseng Innovative Drugs Development, Western Chaoyang Road 45, Changchun 130021, China; 3 Department of Pathogen Biology, Basic Medical College, Jilin University, Changchun 130021, China; wf@jlu.edu.cn

**Keywords:** ginseng, triterpenoids, coagulation factor Xa, inhibitors, thrombin, molecular docking

## Abstract

Enzymes involved in the coagulation process have received great attention as potential targets for the development of oral anti-coagulants. Among these enzymes, coagulation factor Xa (FXa) has remained the center of attention in the last decade. In this study, 16 ginsenosides and two sapogenins were isolated, identified and quantified. To determine the inhibitory potential on FXa, the chromogenic substrates method was used. The assay suggested that compounds **5**, **13** and **18** were mainly responsible for the anti-coagulant effect. Furthermore, these three compounds also possessed high thrombin selectivity in the thrombin inhibition assay. Furthermore, Glide XP from Schrödinger was employed for molecular docking to clarify the interaction between the bioactive compounds and FXa. Therefore, the chemical and biological results indicate that compounds **5** (ginsenoside Rg2), **13** (ginsenoside Rg3) and **18** (protopanaxtriol, PPT) are potential natural inhibitors against FXa.

## 1. Introduction

Thrombosis is a common pathology characterized by the formation of a blood clot inside a blood vessel and the obstruction of blood flow through the circulatory system that underlies three major cardiovascular diseases including acute coronary syndrome, stroke and venous thromboembolism [1]. Venous thromboembolism affects millions of people every year and is responsible for hundreds of thousands of deaths in both the United States and Europe annually [2]. Warfarin, vitamin K antagonists and low-molecular-weight heparins are the most commonly used drugs to reduce thromboembolic occurrence. Despite the prevalence of these anti-coagulants, their employment actually increases the risk of bleeding, brings about the inconvenience of frequent monitoring, interacts with many drugs and foods, as well as has slow onset of action [3], which hinders further clinical application in the treatment of thrombosis. To address the difficulties, more selective anti-coagulants are needed to meet the requirement of efficacy and safety.

Coagulation factor X (FXa) is a vitamin K-dependent serine protease and is one of various enzymes involved in the process of blood coagulation cascade as a catalyst in the conversion of prothrombin to thrombin, which enables FXa to be a potent attractive target for novel anti-coagulant. Compared with the previously prevalent targeting drug thrombin (factor IIa) inhibitors, much evidence has shown that FXa inhibitors were more effective than direct thrombin inhibitors due to FXa’s upstream position from thrombin in the coagulation cascade. Selective inhibition of FXa was unable to affect the pre-existing thrombin level, and activation and aggregation of the platelets reduced the risk of bleeding when compared with traditional anti-coagulants [2]. In addition, FXa plays a vital role in amplifying the process, and is endued with the ability to produce more than one thousand thrombin molecules [2,4]. Furthermore, currently available FXa inhibitors approved by Food and Drug Administration such as rivaroxaban, apixaban and epibaxaban, have been reported to still demonstrate flaws including bleeding risks, narrow clinical applications and drug-drug interactions [2]. Thus, there is a need to develop novel FXa inhibitors with better efficacy and less side-effects.

*Panax ginseng* C. A. Mey, the Araliaceae plant, has been considered as a health food and traditional herbal medicine in Eastern Asia including China, Korea and Bhutan over the past centuries [5]. Nowadays *P. ginseng* is also accessible in small doses in commercial energy beverages or herbal teas. In a previous study, water extracts of *P. ginseng* were reported to possess anti-coagulation effect in vitro [6]. In addition, some Chinese herbal formulas containing ginsenosides also displayed a modulatory effect on the blood coagulation system. For example, a traditional Chinese medicine remedy, Fufang Xueshuantong (including various kinds of ginsenosides) was reported to ameliorate the disorders of the blood coagulation system in a lipopolysaccharide-induced disseminated intravascular coagulation rat model via modulating the activation of the coagulation system [7]. In our previous study, we demonstrated that another traditional Chinese herbal formula Xueshuan Xinmaining Tablets (containing a total ginsenoside of ginseng stems and leaves) enhanced protective activities and anti-oxidative effect of vascular endothelial cells in vitro and removed blood stasis syndrome in murine models, indicating a potential anti-coagulation role in clinical application [8,9]. Furthermore, ginsenosides Rg1 and Rg2 have been reported to possess anti-coagulation properties in vitro [6]. Although *P. ginseng* and part of its major functional components (ginsenosides) have shown anti-coagulation activity in previous studies, whether ginsenosides possess anti-FXa activity and, if so, how ginsenosides interact with surrounding residues have not been reported. Therefore, we designed and conducted a series of anti-coagulation experiments to provide theoretical support for the further development of novel oral-administrated ginsenosides-based FXa inhibitors.

In this study, we aimed to structurally elucidate the components of ginseng, and to determine the content of these ginsenosides through high performance liquid chromatography (HPLC) analysis. Activated partial thromboplastin time (APTT), prothrombin time (PT), and thrombin time (TT) assays were performed to determine the plasma anti-coagulation activity of ginsenosides in vitro, and among ginsenosides demonstrating significant in vitro anti-coagulation effects, the in vitro bioactivities against anti-coagulation factor Xa (FXa) were assessed. Subsequently, ginsenosides that possessed the best bioactivity were molecularly docked with the receptor protein FXa via Schrödinger software to observe the ligand-protein interactions.

## 2. Results

### 2.1. Isolation and Characterization

The purified products above-mentioned were each characterized by NMR analyses. The structures of compounds **1**–**18** were identified as follows: ginsenoside Rg1 (**1**, yield 0.328%), Re (**2**, yield 0.088%), Rf (**3**, yield 0.071%), Rh1 (**4**, yield 0.008%), Rg2 (**5**, yield 0.011%), Rb1 (**6**, yield 0.524%), Rc (**7**, yield 0.121%), Ro (**8**, yield 0.006%), F1 (**9**, yield 0.114%), Rb2 (**10**, yield 0.114%), Rb3 (**11**, yield 0.013%), Rd (**12**, yield 0.123%), Rg3 (**13**, yield 0.021%), 20(*R*)-Rg3 (**14**, yield 0.001%), Rh2 (**15**, yield 0.001%), F2 (**16**, yield 0.001%), protopanaxdiol (PPD, **17**, yield 0.071%), protopanaxtriol (PPT, **18**, yield 0.001%) [10,11,12,13,14,15,16,17,18].

### 2.2. Determination of Content for the Compounds from Ginseng

The dried ginseng root powder (1.0 g, 60 mesh sieve) was accurately weighed and was added to two volumes of water. The mixture was heated under 100 °C for 2 h. Next, the filtration was added to a D101 macroporous adsorption resin column eluted with water and 80% ethanol consecutively. The 80% ethanol elution was concentrated to the residue, which was dissolved in 10 mL of methanol.

Some standard compounds were also used in this study. Ginsenoside Rg1 (110703-200726), ginsenoside Re (110754-200822), ginsenoside Rb2 (111715-201203), ginsenoside Rb3 (111686-201203), ginsenoside Rd (111818-201302), ginsenoside Rg3 (110804-201504), ginsenoside Rf (111719-201505), ginsenoside Rh2 (111748-200501), ginsenoside Rg2 (111779-200801), ginsenoside Ro (111903-201604), protopanaxdiol (111747-200501) and protopanaxtriol (111755-200601) were purchased from the National Institutes for Food and Drug Control (Beijing, China). Ginsenosides Rh1, Rb1, Rc, F1, 20(*R*)-Rg3 and F2 were provided by the New Drug Research and Development Laboratory of Jilin University.

**Figure 1:**
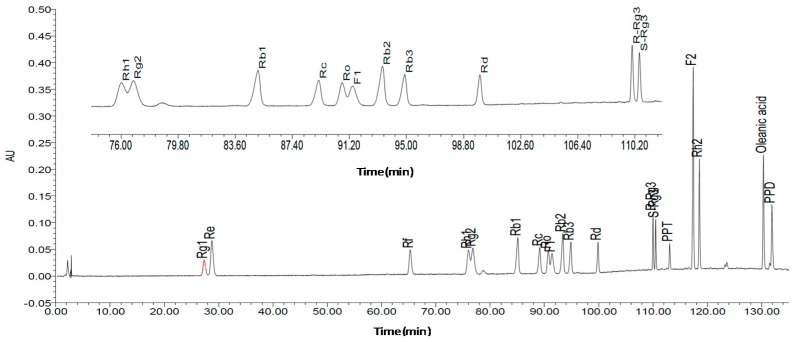
High performance liquid chromatography (HPLC) record of mixture of standards.

**Figure 2:**
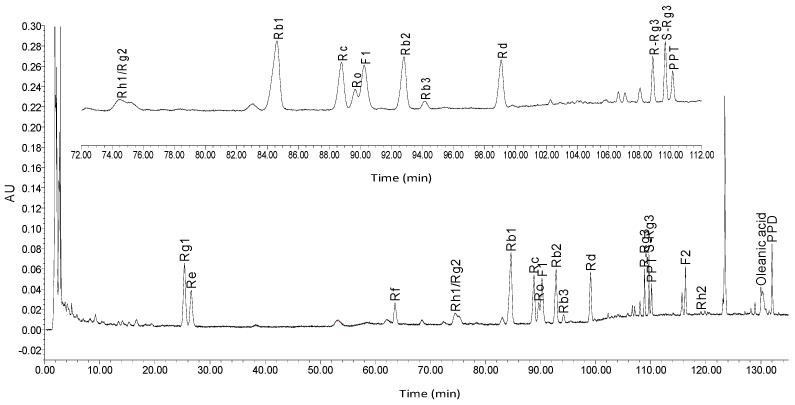
HPLC record of ginseng.

The determination of the saponins was performed using a HPLC system. The detection wavelength was 203 nm. Column temperature was 40 °C. The mobile phase was comprised of acetonitrile (A) and 1% acetic acid in water solvent (B). The gradient mode was as follows: initial 20% A linear gradient to 22% A in 25 min; linear gradient to 28% A in 55 min; linear gradient to 35% A in 95 min; linear gradient to 60% A in 112 min; and linear gradient to 100% A in 135 min. The flow rate was 1.3 mL/min. The components were identified through comparison of the retention time from the chromatograms with known standards. The contents of Rg1, Re, Rf, Rh1 + Rg2, Rb1, Rc, Ro, F1, Rb2, Rb3, Rd, Rg3, 20(*R*)-Rg3, Rh2, F2, PPD and PPT were 0.4659%, 0.1061%, 0.0908%, 0.0230%, 0.6145%, 0.1412%, 0.0017%, 0.1581%, 0.1497%, 0.0152%, 0.1546%, 0.0021%, 0.0342%, 0.0001%, 0.0001%, 0.0001%, 0.0377% and 0.0958%, respectively. The fingerprints of the mixed standard compounds and the extract of ginseng are shown in Figure 1 and Figure 2.

### 2.3. In Vitro Effects of Ginsenosides on Human Blood Clotting Time

**Figure 3:**
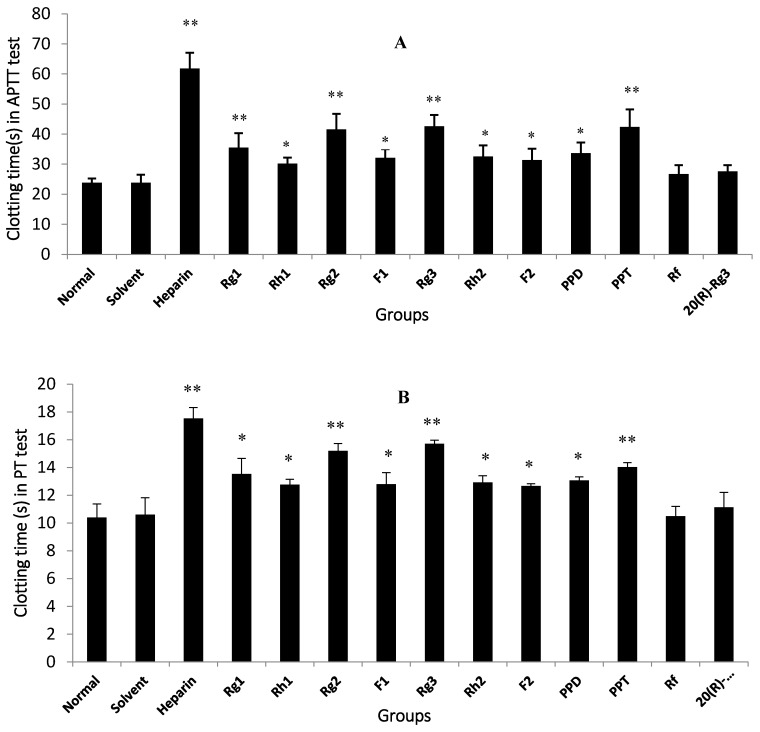

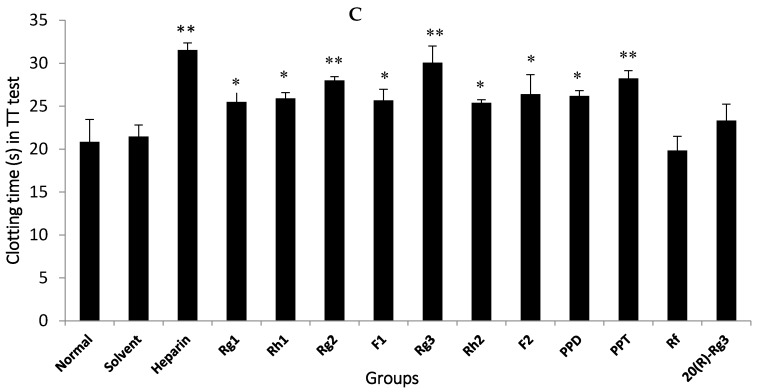
In vitro anti-coagulation activities of 11 ginsenosides. (**A**) APTT test; (**B**) PT test; (**C**) TT test. * *p* < 0.05, ** *p* < 0.01 versus the normal control. APTT: activated partial thromboplastin time; PT: prothrombin time; TT: thrombin time.

At final concentrations of 0.05 mg/mL, nine out of 18 ginseonsides showed significant anti-coagulant effects in vitro compared to the normal control (*p* < 0.05) (Figure 3) and were selected to be further detected for their anti-FXa activity in vitro. Among the nine ginsenosides with excellent anti-coagulation activity in vitro, Rg2, Rg3 and PPT possessed the best bioactivity (*p* < 0.01) and other ginsenosides including Rg1, Rh1, F1, Rh2, F2 and PPD showed significant anti-coagulation activity compared to the normal control (*p* < 0.05) in APTT, PT and TT tests. In contrast, the solvent group, Rf and 20(*R*)-Rg3 displayed insignificant anti-coagulation effects when compared to the normal control in all three coagulation parameters (*p* > 0.05).

### 2.4. Effects of Ginsenosides on FXa Activities In Vitro

**Figure 4:**
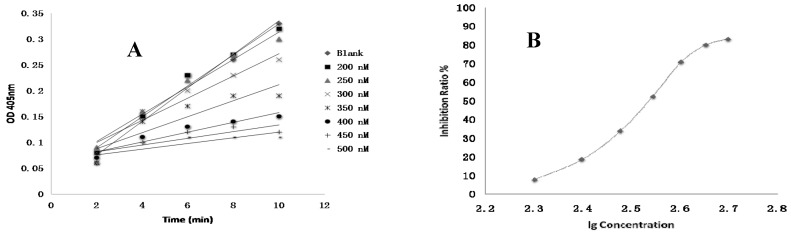

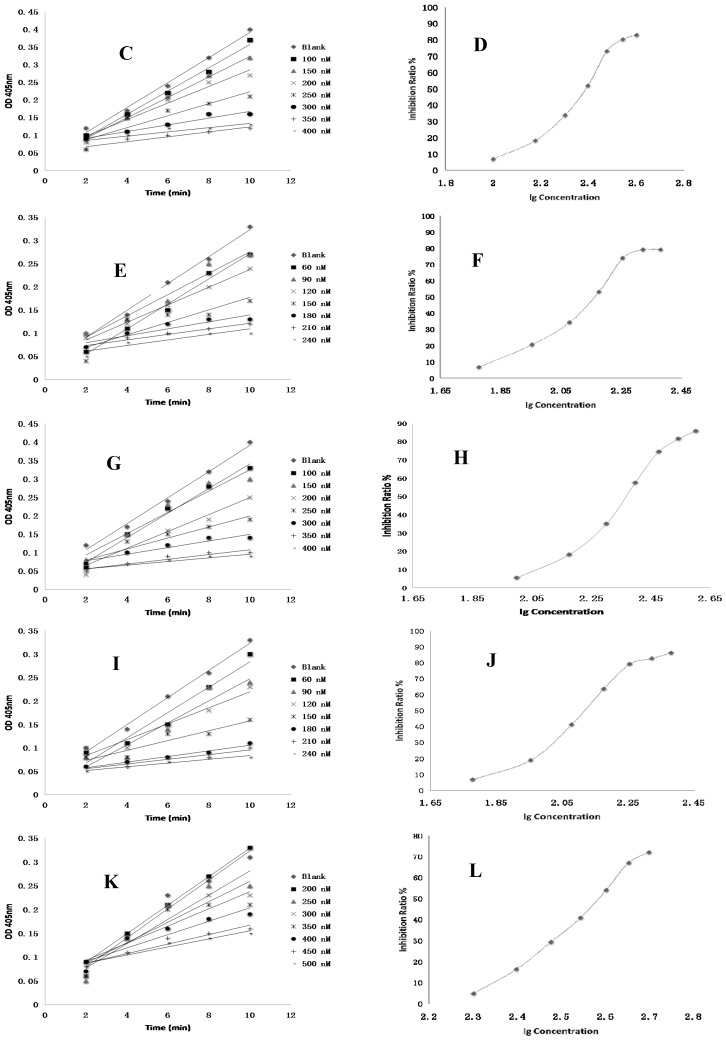

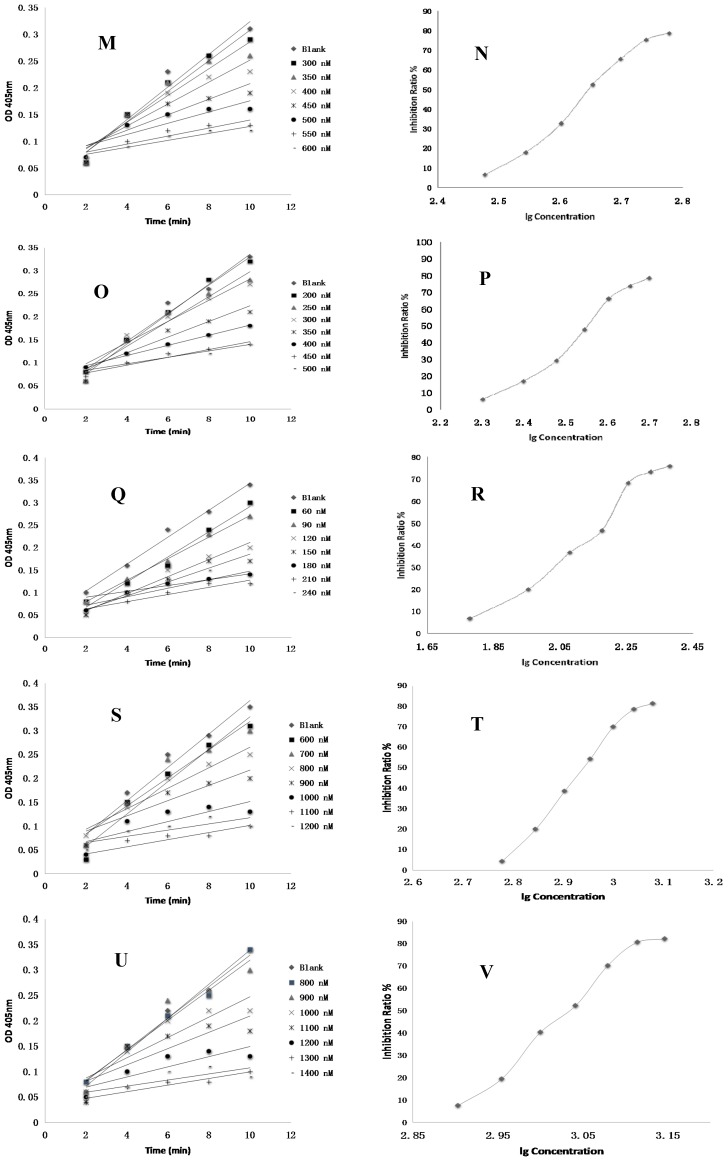

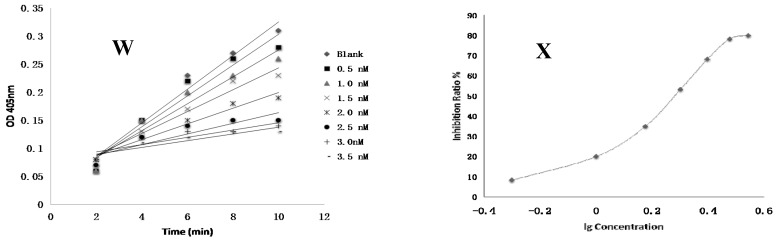
The inhibition profile figures against coagulation factor X. (**A**,**B**) Rg1; (**C**,**D**) Rh1; (**E**,**F**) Rg2; (**G**,**H**) F1; (**I**,**J**) Rg3; (**K**,**L**) Rh2; (**M**,**N**) F2; (**O**,**P**) PPD; (**Q**,**R**) PPT; (**S**,**T**) 20(R)-Rg3; (**U**,**V**) Rf; (**W**,**X**) Rivaroxaban. lg: log^10^, OD: optical density.

Among the nine ginsenosides showing excellent anti-coagulant activities and the two ineffective ginsenosides in vitro, ginsenosides Rg2, Rg3 and PPT exhibited the best anti-FXa activities with fifty percent of inhibitory concentration (IC_50_) of 135.9, 126.7 and 140.7 nM, respectively (Table 1). The positive control drug showed higher activity with IC_50_ of 1.9 nM compared to all test ginsenosides (Figure 4).

### 2.5. Selectivity Versus Thrombin

**Figure 5:**
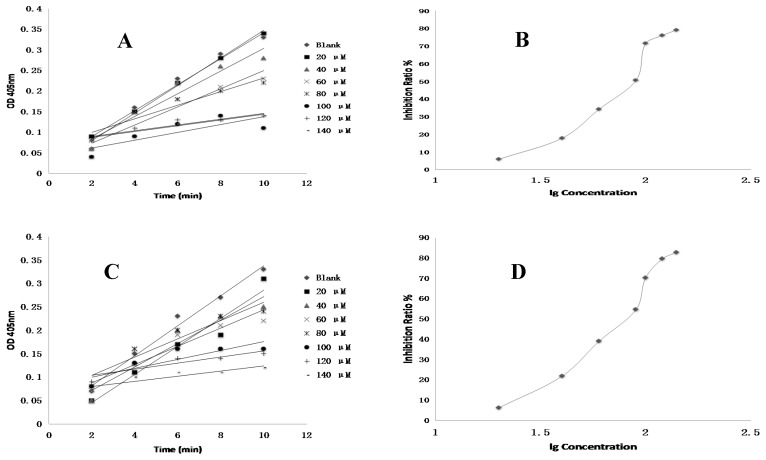

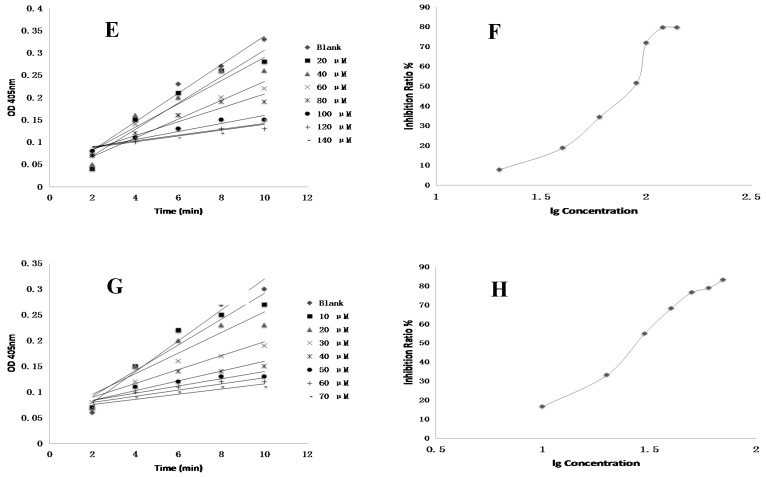
The inhibition profile figures of ginsenoside (Rg2) (**A**,**B**); ginsenoside (Rg3) (**C**,**D**); protopanaxtriol (PPT) (**E**,**F**) and Ximelagatran (**G**,**H**) against thrombin.

In the previous anti-coagulant assay in vitro, Rg2, Rg3 and PPT demonstrated excellent FXa inhibitory activity and were further selected to assess their selectivity versus thrombin. Rg2, Rg3 and PPT displayed a high level of selectivity against thrombin with IC_50_ values of 81.3, 92.6, 82.0 μM, respectively, which were higher than that of ximelagatran with IC_50_ values of 27.1 μM (Table 2, Figure 5). The evidence showed that Rg3 showed the best anti-coagulant activity in vitro and also demonstrated the highest selectivity against thrombin.

### 2.6. Interactions between Ginsenosides and FXa Protein

**Figure 6:**
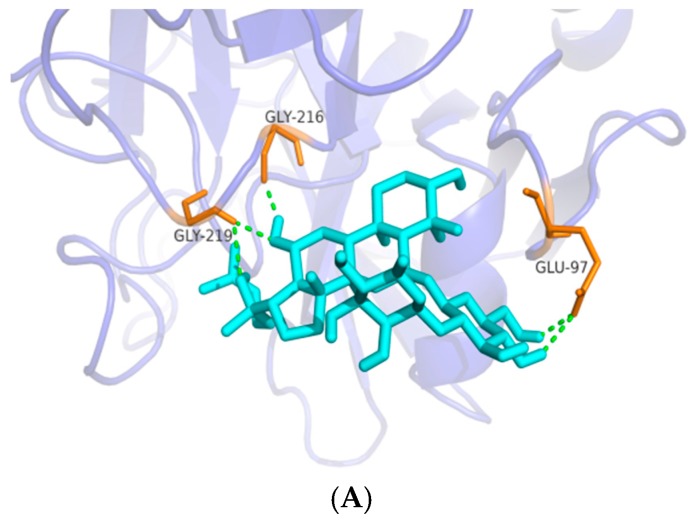

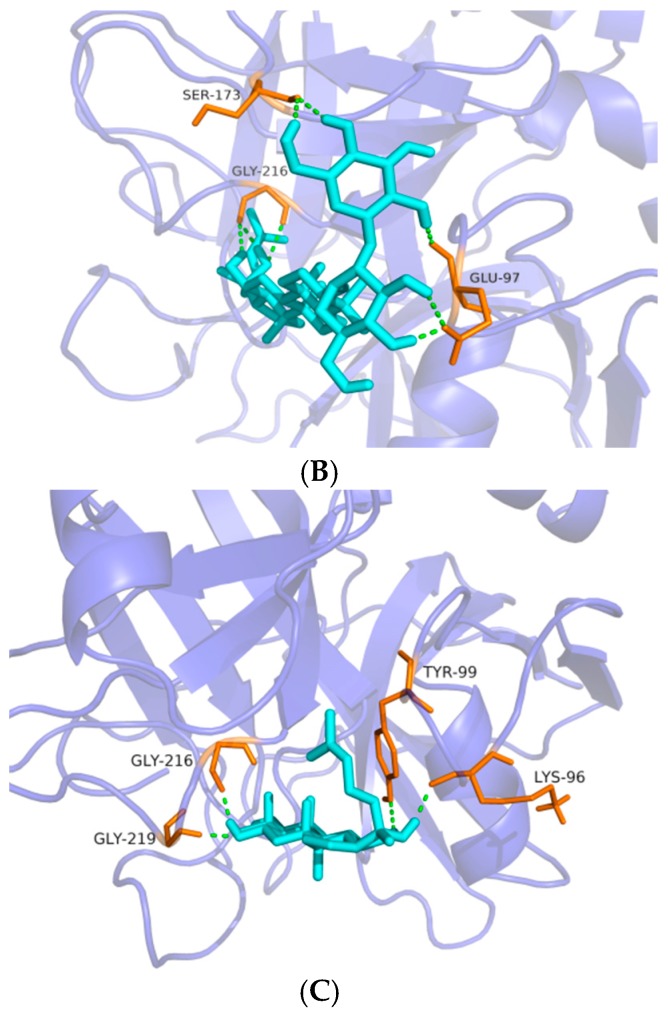
Interaction modes of ginsenosides **5**, **13** and **18** within FXa binding pocket (**A**–**C**). (**A**) H-bonds between Rg2 (**5**) and FXa pocket; (**B**) H-bonds between Rg3 (**13**) and FXa pocket; (**C**) H-bonds between PPT (**18**) and FXa pocket; light blue, ligands (Rg2, Rg3 and PPT); pink, ligands; orange, residues of the binding protein (FXa); green dashed line, H-bond.

Molecular docking investigation was performed to clarify the interaction modes of the most active ginsenosides in an anti-coagulation assay on FXa protein and to measure the relative binding energies and localize accurate binding sites in the active pocket. As shown in Figure 6, Rg3 showed the most hydrogen bonds with nearby residues among the best anti-coagulation ginsenosides in bioactivity assays. Among the binding amino acid residues between ligands and the protein, GLY-216 was commonly shared among the three ginsenosides. Furthermore, the same residue GLU-97 was shared by ligands Rg2 and Rg3.

## 3. Discussion

The anti-coagulation effects of 18 compounds were investigated, among which Rg1, Rg2, Rg3, Rh1, F1, Rh2, F2, PPD and PPT possessed significant anti-coagulation activities in vitro compared to the normal control (*p* < 0.05). Next, the Rg2, Rg3 and PPT that displayed the best anti-FXa activity (*p* < 0.01) were further molecularly docked with human FXa protein. The result by Glide XP from Schrödinger revealed the interactions and accurate binding sites between the bioactive FXa and the three ginsenosides, which further supported the data of the bioactivity assays and also pointed out the potential future direction of chemical modification. All the above-mentioned evidence indicates that Rg2, Rg3 and PPT may be potential natural FXa inhibitors.

In a previous study of the water extract of *P. ginseng*, together with water extracts of the same family (Araliaceae genus *Panax quinquefolius* L. and *Panax notoginseng* (Burk.) F.H. Chen), it was observed to significantly extend blood clotting time of activated partial thromboplastin, prothrombin and thrombin in in vitro human plasma coagulation assays [6]. Similarly, the ethyl acetate fraction of Korean red ginseng methanol extracts showed potent anti-coagulant activity via markedly prolonged clotting time that was measured by thrombin time [19]. In another report, 0.05 mg/mL (final concentration) Rg1 and Rg2 exhibited significantly better anti-coagulant activities in vitro compared to 0.1 mg/mL (final concentration) heparin that was chosen as the positive control drug [20]. In the present study, nine ginsenosides exhibited significant anti-coagulation activity (Figure 3), which was consistent with the previous findings. However, not all evidence supported the anti-coagulation role of ginsenosides in the blood coagulation cascade. In contrast to our data, Wee et al. [19] observed that ginsenosides did not inhibit the blood coagulation process. In this investigation, a 50% MeOH subfraction demonstrated higher potent inhibition against blood coagulation compared to the 100% MeOH subfraction; nevertheless, ginsenosides Rf, Rh1 and Rg3 that were present in the EtOAc fraction were weakly detected in the 50% MeOH subfraction, indicating that saponins did not contribute to the anticoagulation activity of the EtOAc fraction. In the current study, nine ginsenosides that showed significant anti-coagulation activity in vitro were subsequently studied for their anti-FXa effect in vitro.

FXa inhibitors have emerged as new anti-coagulant drugs and when compared to previous anti-coagulants, they possess the following advantages: more rapid onset and offset of action; reduction in need for “bridging” with a parenteral anti-coagulant; less clinical monitoring; and less interactions with other drugs and food. However, for current FXa inhibitors there are still a few drawbacks, such as a lack of antidotes, short half-life affecting efficacy, as well as high acquisition costs which limits wider clinical application. Therefore, natural occurring FXa inhibitors may be an ideal substitute. Many studies have revealed that natural polypeptides isolated from organisms such as bloodsuckers and *Ancylostoma caninum* could act as direct inhibitors against factor Xa [21]. However, due to the near impossibility of these polypeptides being developed for oral administration, attention may focus instead on natural small molecular compounds. In a study published in 2014, a natural small molecule (glycyrrhetinic acid) derived from the Chinese herb *Glycyrrhiza glabra* was described in [21]. The orally administered compound was reported to significantly inhibit FXa activity in vitro with specificity, and reduce thrombus weight in rat models despite the unequalable anticoagulation property with commercially accessible drugs rivaroxaban and apixaban, suggesting that glycyrrhetinic acid could be considered as a promising natural FXa inhibitor [21,22]. In this study, ginsenosides that mainly originate from herbs of the *Panax* species and possess a molecular weight less than 1000 demonstrated significant inhibitory activities against FXa in vitro. Among the nine ginsenosides with significant anti-coagulation effects in vitro, Rg2, Rg3 and PPT showed the highest affinity with FXa receptors (Table 1 and Figure 4), and also displayed excellent thrombin selectivity (Table 2). FXa is structurally similar tothrombin (coagulation factor II), which may result in the binding of FXa inhibitors to thrombin and subsequent risk of bleeding. Furthermore, selectivity is an important issue for the development of FXa inhibitors [2]. Hence, there is a need to address the selectivity issue for the development of newer FXa inhibitors by understanding structural differences between FXa and thrombin, and for the reason we designed and conducted the experiment to test whether ginsenosides showing significant anti-FXa activity were unable to affect thrombin level. The data showed that Rg2, Rg3 and PPT were potent FXa inhibitors with high thrombin selectivity, indicating that these three ginsenosides may be potent anti-coagulants with less risk of bleeding. Further molecular docking by Schrödinger software confirmed the data obtained in the anti-coagulation activity assays and pointed out the specific binding protein residues around the active pocket, which may form the basis for future chemical modification in ginsenosides.

Despite our finding that micromolecule direct FXa inhibitors in ginseng was accomplished in the anti-coagulation assay in vitro, and that anti-FXa and thrombin selectivity assays may reveal potent anti-coagulation activity in the future treatment of coagulation-associated disorders, nevertheless, there are some unresolved issues. First, the phenomenon that multiple ginsenosides possess anti-coagulation properties have been reported in the current research; however, the underlying action mechanism of the anti-coagulation effect of ginsenosides still remains unclarified and requires further investigation. Additionally, the unstable chemical structure of ginsenosides such as Rg3 under acidic and high-temperature conditions, especially in the stomach, may hinder its further application in scientific research and trials [20], furthermore, ginsenosides have demonstrated incomparable bioactivity with the positive drug heparin in an anti-coagulation assay in vitro and rivaroxaban in an anti-FXa test in vitro. Therefore, to develop novel anti-coagulants with better efficacy and lower side effects, future work is required.

## 4. Materials and Methods

### 4.1. General

A Bruker DRX 500 spectrometer (Bruker Biospin, Rheinstetten, Germany), using tetramethylsilane (TMS) as an internal standard, was used for NMR spectra. The qualitative, quantitative analyses and HPLC separations were all performed on a Waters 1525 Binary HPLC Pump equipped with Waters 2998 Photodiode Array Detector (Waters Corporation, Milford, MA, USA). SunFire Prep C18 Column (10 mm × 150 mm, silica gel particle size: 10 μm) was used for HPLC separation and Diamonsil C18 (4.6 mm × 250 mm, 5 μm) was used for HPLC analyses. Silica gel for column chromatography was provided by the Qingdao Ocean Chemical Group Co. (Qingdao, China). Pyridine-*d*_5_ was purchased from the Sigma-Aldrich Company (St. Louis, MO, USA). For plasma preparation, the micro-centrifuge was obtained from Thermo Fisher Scientific (Heraeus Fresco 21, Harz, Germany). For blood clotting time assays, a coagulometer was purchased from Beckman Coulter Inc. (Beckman ACL TOP-700, CA, USA), and the positive control drug heparin was obtained from YM Biological Technology Company Limited. For the anti-FXa activity assay, purified FXa and FXa substrate CS-11(22) were purchased from New England Biolabs (Hitchin, UK) and American Diagonostica Inc. (Stamford, CT, USA), respectively. An automated microplate reader Epoch was obtained from Bio-Tek Instruments Inc. (Winooski, VT, USA) and the positive drug rivaroxaban was obtained from Bayer Health Care AG (Wupertal, Germany). For the thrombin selectivity assay, FIIa and chromogenic substrate CS-01(38) were purchased from Hyphen BioMed (Paris, France), and the positive drug ximelagatran was purchased from AstraZeneca LP (Molndal, Sweden). For the anti-coagulation activity assay in vitro, the solvent dimethyl sulfoxide (DMSO) and heparin sodium salt were obtained from Sigma-Aldrich Company (St. Louis, MO, USA). For data analysis, the software SPSS 19.0 was obtained from SPSS Inc. (Chicago, IL, USA).

### 4.2. Plant Material

*Ginseng quinquennium*, was collected in Ji’an City in Jilin Province, China, in August 2015. The plant material was identified by Professor. Pingya Li (School of Pharmaceutical Sciences, Jilin University). A voucher specimen (No. 20150928) was deposited in the School of Pharmaceutical Sciences, Jilin University.

### 4.3. Preparation of the Total Saponins of Ginseng

The powdered air-dried root of ginseng (300 g) was extracted with 70% ethanol under reflux for 3 h and repeated for three times. The extracted solution was then concentrated under reduced pressure. The extract (63 g) was purified in an AB-8 macroporous adsorption resin column, eluted with water, 20% ethanol and 85% ethanol, respectively. Finally, the 85% ethanol elute (25.1 g) was collected as the total saponins from ginseng.

### 4.4. Extraction and Isolation

The total saponins from ginseng were subjected to column chromatography in a silica gel column gradiently eluted with chloroform/methanol repeatedly to give eight fractions, Fraction **A** (chloroform/methanol (100:4, *v*/*v*)), Fraction **B** (chloroform/methanol (100:8, *v*/*v*)), Fraction **C** (chloroform/methanol (100:9, *v*/*v*)), Fraction **D** (chloroform/methanol(100:12, *v*/*v*)), Fraction **E** (chloroform/methanol (100:15, *v*/*v*)), Fraction **F** (chloroform/methanol (100:18, *v*/*v*)), Fraction **G** (chloroform/methanol (100:20, *v*/*v*)), and Fraction **H** (chloroform/methanol (100:30, *v*/*v*)). Compounds **17** and **18** were recrystallized from Fraction **A** using ethyl acetate. Compound **15** was purified from Fraction **B** by using reverse phase silica gel chromatography eluted with methanol/water gradiently. Fraction **C** was purified with octadecyl-bonded silica gel (ODS) column eluted with methanol/water (55:45, *v*/*v*) to obtain compounds **4** and **9**. Compound **5** was obtained from Fraction **D** using column chromatography on silica gel repeatedly using acetone and ethyl acetate. Fraction **E** was separated on normal phase (using a mixture of chloroform, ethyl acetate, methanol and water as elution) and reverse phase (using gradient methanol/water as elution) silica gel column chromatography to obtain compounds **1**, **3**, **13**, **14** and **16**. Compounds **7**, **2** and **12** were purified from Fraction **F** by column chromatography on silica gel repeatedly using dichloromethane and methanol, respectively. Compounds **10** and **11** were yielded from Fraction **G** by column chromatography on ODS, eluted using gradient methanol and water. Fraction **H** was separated in reverse phase (using gradient methanol/water as elution) silica gel column chromatography to obtain compounds **6** and **8**.

### 4.5. Animal Preparation

Male Sprague-Dawley rats weighing 180–220 g were purchased from the Animal Center of Norman Bethune Medical College of Jilin University in China. The rats were maintained under controlled environment (22 ± 2 °C, relative humidity 40–60%, 24 h light-dark cycles, and ad libitum access to food and water). The animal experiments were conducted based on the guide for the administration of laboratory animals (Directive 86/609/EEC in the Protection of Animals Used for Experimental and Other Scientific Purposes, 1986) and were approved by the Institutional Animal Care and Use Committee (IACUC) of Jilin University (No. SCXK-2013-0001).

### 4.6. Blood Collection and Preparation of Plasma Samples

Rats were anaesthetized with 10% chloral hydrate (3 mL/kg). The blood obtained from the abdominal aorta was collected directly into citrated tubes containing 3.8% sodium citrate (1:9 (*v*/*v*)) and was used immediately after collection [23]. Platelet poor plasma (PRP) was prepared by centrifuging the blood at 3000 rpm for 20 min at 20 °C [24]. Plasma samples with jaundice, chylus, hemolysis and blood clot were excluded prior to the assays. Plasma mixtures were 90 μL of PRP with 10 μL of tested compounds (0.5 mg/mL, dissolved in DMSO). For PRP in the solvent group, the sample was 90 μL of PRP with 10 μL of DMSO. For PRP in the normal control group, the plasma was not-treated PRP sample. For PRP in the heparin group, the sample was 90 μL of PRP with 10 μL of heparin (0.1 mg/mL), which was used as the positive control drug.

### 4.7. Measurement of Blood Clotting Time

Blood coagulation assays were carried out using a coagulation analyzer at the Blood Center clinical laboratory in the Second Hospital affiliated to Jilin University (Changchun, China). Measurements of APTT, PT and TT were performed according to the manufacturer’s recommended protocols. The anti-coagulant activity was expressed as clotting time ± standard deviation (S.D.). Briefly, for APTT assay, 50 μL of the plasma mixture was incubated at 37 °C for 60 s and the mixture was added to the APTT reagent (50 μL) and incubated at 37 °C for 15 min. Finally, APTT values were recorded. For the PT assay, 50 μL of the plasma mixture was incubated at 37 °C for 60 s and then added into the PT reagent (100 μL) prior to incubation at 37 °C for 15 min before the PT values were recorded. For the TT assay, 80 μL of the plasma mixture was incubated at 37 °C for 60 s before the addition of 80 μL of TT reagent for 100 s. TT values were determined by the coagulation analyzer.

### 4.8. FXa Activity Assay of Compounds In Vitro

Test compounds and the positive control drug rivaroxaban were dissolved in DMSO at a concentration of 1 mM and then serially diluted to a range of 3 nM to 10 µM, respectively. 10 µL of FXa (final concentration of 0.5 nM), 40 µL of Tris buffer (adjusted to pH 7.4 with HCl containing 0.3 M NaCl and 50 mM Tris) and 10 µL of test compounds were added to the well, respectively. The negative control was performed using the same mixed solutions except that the test compound was replaced with DMSO. The positive control was composed of the same mixed solutions except that the test compound was replaced with rivaroxaban. After 15 min of incubation at 37 °C, FXa substrate (40 μL, final concentration of 0.25 nM) was added and then was incubated 37 °C for 25 min [3]. The optical density (OD) values at 405 nm were evaluated by an automated microplate reader. The time-absorbance curve and the slope of curve reflecting enzymatic activity were observed in test groups (*V_i_*), positive control group (*V_i_*) and negative control group (*V*_0_). Inhibition rate was calculated by the following formula: Anti-FXa activity = (*V*_0_ − *V_i_*)/*V*_0_ [21]. The IC_50_ value was subsequently calculated by SPSS 19.0.

### 4.9. Thrombin Inhibition In Vitro of Compounds

The inhibition of thrombin was evaluated by human FIIa and chromogenic substrate CS-01(38) in 96-well microtiter plates at room temperature. Compounds **5**, **13** and **18** as well as the positive reagent Ximelagatran were dissolved in DMSO to a concentration of 1mM and then serially diluted to a range of 10 μM to 100 μM, respectively. 8 μL of FIIa (3 NIH U/mL), 80 μL of Tris buffer (adjusted to pH 7.4 with HCl) containing 0.3 M NaCl and 50 mM Tris and 8 μL of test compounds were added to the well, respectively. The negative control was performed using the same mixed solutions except that tested compounds were replaced with DMSO. The positive control was performed using the same mixed solutions except that the test compounds were replaced with ximelagatran. After incubation at 37 °C for 15 min, 12 μL of FIIa substrate solution (4 mM) was added and then incubated at 37 °C for 25 min [3]. The anti-FIIa activity was measured at 405 nm using a microplate reader.

### 4.10. Molecular Docking of Rg2, Rg3 and PPT within FXa

Molecular docking study of bioactive compounds was performed using GLIDE (Grid-based Ligand Docking with Energetics) (GLIDE, version 6.7, Schrödinger, LLC, New York, NY, USA) software developed by Schrödinger. Maestro (version 2015-2, Schrödinger, LLC, New York, NY, USA) was used for all the steps involving protein and ligand preparation, receptor grid generation and docking. The X-ray crystal structure of FXa (Protein Data Bank (PDB) code: 2w26) complexed with an oral and direct FXa inhibitor Bay59-7939 was retrieved from the PDB database (http://www.rcsb.org/pdb) based on a previous study [2]. The Protein Preparation Wizard in the GLIDE software was used to prepare the receptor FXa. The structure of FXa was optimized after a series of processes including assigning bond orders and water orientations, removing water molecules, adding hydrogens, creating zero-order bonds to metals and disulfide bonds. The protein was then energy minimized using a default constraint of 0.30 Å root-mean-square deviation (RMSD) using the optimized potentials for liquid simulations 3 (OPLS3) force field. When performing receptor grid generation, a present ligand in the retrieved protein-ligand complex was identified prior to setting the center and the size of the box. The grid box was limited to the size of 20 Å at the active site. Crystal coordinates of compounds (ligands) were pre-drawn in Maestro Elements (Maestro Elements, version 2.2, Schrödinger, LLC, New York, NY, USA) prior to the molecular docking study. Three-dimensional (3D) structures of all 18 compounds were generated using LigPrep module (2015-2) from the Schrödinger Suite (LLC) by assigning the bond orders and angles. In addition, these compounds were subjected to minimization using the OPLS3 force field. For GLIDE docking, the prepared structure of FXa and ligands (compounds) were imported to the workspace using GLIDE v.6.7 from the Schrödinger Suite [25,26,27]. Extra precision (XP) docking was carried out and the parameters of scaling factor and partial charge cutoff were set at the default values 0.80 and 0.15, respectively. At least the top ten ranking conformations for each ligand was chosen in the output tab to set the output numbers. Figures of the docking results were subsequently prepared using PyMol (Schrödinger).

### 4.11. Statistical Analysis

All values were expressed as means ± standard deviation (SD), and one-way analysis of variance (ANOVA) and student’s *t*-test were performed by SPSS19.0 (SPSS Inc., Chicago, IL, USA). *p* < 0.05 was considered statistically significant.

## 5. Conclusions

In summary, triterpenoids from ginseng are potential natural coagulation factor Xa (FXa)-inhibitors with high thrombin selectivity and prolongation of coagulation time. The bioactivity studies and HPLC analysis also suggested that despite the low content in total saponins, the three triterpenoids, Rg2 (**5**, 0.0230%), Rg3 (**13**, 0.0021%) and PPT (**18**, 0.0958%), maybe responsible for the anti-coagulant effect. Hence, the total saponins and the effective components could be used as a potential natural anticoagulation therapy.

## Figures and Tables

**Table 1:**
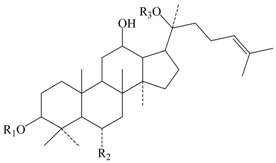
The structures of 11 ginsenosides and their anticoagulation activities against coagulation factor Xa (FXa).

Compound	R_1_	R_2_	R_3_	IC_50_ (nM)
Rg1	H	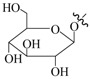	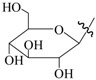	334.7
Rh1	H	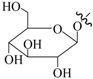	H	235.8
Rg2	H	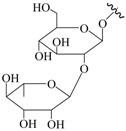	H	135.9
F1	H	H	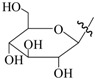	227.0
Rg3	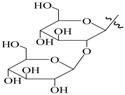	H	H	126.7
Rh2	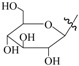	H	H	348.7
F2	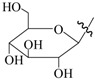	H	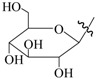	425.2
PPD	H	H	H	339.4
PPT	H	OH	H	140.7
20(*R*)-Rg3	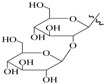	H	H	815.3
Rf	H	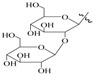	H	1034.0
Rivaroxaban				1.9

IC_50_: fifty percent of inhibitory concentration; PPD: protopanaxdiol; PPT: protopanaxtriol.

**Table 2:** Selectivity versus thrombin.

Compound	Thrombin IC_50_ (μM)
Rg2	81.3
Rg3	92.6
PPT	82.0
Ximelagatran	27.1

## Chemical Compounds

Rg1 (PubChem CID: 441923)
Re (PubChem CID: 441921)
Rf (PubChem CID: 441922)
Rh1 (PubChem CID: 122130363)
Rg2 (PubChem CID: 12912322)
Rb1 (PubChem CID: 122130642)
Rc (PubChem CID: 122130031)
Ro (PubChem CID: 11815492)
F1 (PubChem CID: 9809542)
Rb2 (PubChem CID: 6917976)
Rb3 (PubChem CID: 12912363)
Rd (PubChem CID: 11679800)
Rg3 (PubChem CID: 9918693)
20(*R*)-Rg3 (PubChem CID: 46887680)
Rh2 (PubChem CID: 119307)
F2 (PubChem CID: 9918692)
protopanaxdiol (PubChem CID: 9920281)
protopanaxtriol (PubChem CID: 22392424).
